# Colonization of toxigenic *Clostridium difficile* among ICU patients: a prospective study

**DOI:** 10.1186/s12879-016-1729-2

**Published:** 2016-08-09

**Authors:** Xiaoxia Zhang, Xiaohui Wang, Jingyu Yang, Xiaohua Liu, Lin Cai, Zhiyong Zong

**Affiliations:** 1 Center of Infectious Diseases, West China Hospital, Sichuan University, Guoxuexiang 37, Chengdu, 610041 China; 2 Division of Infectious Diseases, State Key Laboratory of Biotherapy, Chengdu, China; 3 Department of Infection Control, West China Hospital, Sichuan University, Chengdu, China; 4 Intensive Care Unit, West China Hospital, Sichuan University, Chengdu, China; 5 Department of Clinical Microbiology, Xindu District Hospital, Chengdu, China

**Keywords:** *Clostridium difficile*, Colonization, Active screening, ICU, MLST, MLVA

## Abstract

**Background:**

A prospective study was performed to investigate the prevalence of colonization among ICU patients and to examine whether asymptomatic carriers were the source of subsequent *C. difficile* infection (CDI) and acquisition of toxigenic *C. difficile*.

**Methods:**

Rectal swabs were collected from adult patients on admission to and at discharge from a 50-bed medical ICU of a major referral hospital in western China, from August to November 2014. Stools were collected from patients who developed ICU-onset diarrhea. Both swabs and stools were screened for *tcdB* (toxin B gene) by PCR. Samples positive to *tcdB* were cultured for *C. difficile* and isolates recovered were screened for *tcdB* and the binary toxin genes by PCR. Strain typing was performed using multilocus sequence typing and isolates belonging to the same sequence type (ST) were further typed using multiple-locus variable number tandem repeat analysis (MLVA).

**Results:**

During the 4-month period, rectal swabs were collected from 360 (90.9 %) out of 396 patients who were admitted to the ICU. Among the 360 patients, 314 had stayed in the ICU more than 3 days, of which 213 (73.6 %) had a rectal swab collected within the 3 days prior to discharge from ICU. The prevalence of toxigenic *C. difficile* colonization was 1.7 % (6 cases) and 4.3 % (10 cases) on admission and discharge, respectively. Only four (1.1 %) out of 360 patients had CDI, corresponding to 10.7 cases per 10,000 ICU days. None of the four cases had toxigenic *C. difficile* either on admission or at discharge. Toxigenic *C. difficile* isolates were recovered from all swabs and stool samples positive for *tcdB* by PCR and belonged to 7 STs (ST2, 3, 6, 37, 54, 103 and 129). None of the isolates belonging to the same ST had identical MLVA patterns. Binary toxin genes were detected in one ST103 isolate that caused colonization.

**Conclusion:**

The prevalence of colonization with toxigenic *C. difficile* among patients on admission to ICU was low in our setting. ICU-acquired toxigenic *C. difficile* were not linked to those detected on admission. Active screening for toxigenic *C. difficile* may not be a resource-efficient measure in settings with a low prevalence of colonization.

## Background

Toxigenic *Clostridium difficile* has been well recognized as a major pathogen causing healthcare-associated infections in western countries [1]. In healthcare settings, *C. difficile* can be transmitted via contact with patients or their environment directly or indirectly. Contact precautions are therefore required when *C. difficile* infection (CDI) is suspected [2, 3]. Some patients can be colonized with toxigenic *C. difficile* but without manifestations of CDI. These asymptomatic carriers may serve as an important reservoir of toxigenic *C. difficile* [4] and appear to have significant higher risks of subsequent CDI [5]. Active screening patients for toxigenic *C. difficile* may therefore have two benefits. One is to identify the source of this pathogen for subsequent control measures, such as contact precautions for carriers, and another is to identify the population at high risk of CDI. However, active screening is resource-intensive and requires corresponding laboratory capacity, which may be problematic in developing countries like China. In settings with a high prevalence of colonization with toxigenic *C. difficile* and where nosocomial transmission is frequently linked to asymptomatic carriers, it may be justified to include active screening in the prevention and control of CDI. There are a few studies on the prevalence of colonization for inpatients and residents in long-term care facilities or nursing homes [5, 6]. However, colonization with toxigenic *C. difficile* among ICU patients remains largely uninvestigated. In addition, little is known about the carriage and transmission of toxigenic *C. difficile* in China. We therefore performed a prospective study to investigate the prevalence of colonization with toxigenic *C. difficile* among patients in a large ICU of a major teaching hospital in China and to examine whether the toxigenic *C. difficile* isolates acquired during the ICU stay were linked to asymptomatic carriers who were colonized on admission to the unit.

## Methods

### Patient enrollment and sample collection

This prospective study was conducted among adult patients (age ≥16) at a 50-bed medical ICU ward in West China Hospital, Sichuan University, the major referral medical center in western China, during a 4-month period from August to November 2014. This study was conducted in accordance with the amended Declaration of Helsinki and was approved, under a waiver of consent, by the Ethics Committee of West China Hospital. Stool samples and rectal swabs were taken as part of routine care as collecting stool specimens or rectal swabs for screening multidrug resistant organisms such as extended-spectrum β-lactamase-producing Enterobacteriaceae and vancomycin-resistant enterococci has been a routine practice in the ICU.

Rectal swabs were collected from patients within 2 days of admission to the ICU and also within the 3 days prior to ICU discharge for those patients with a length of stay of 3 days or more. Swabs were transferred to the laboratory in transport media. Stool samples were also collected from patients who developed ICU-onset diarrhea with naturally-passed faeces that were defined as unformed at least 3 times a day.

The presence of toxigenic *C. difficile* in a rectal swab or stool sample was detected by PCR for the toxin B-encoding gene *tcdB* of *C. difficile* (see below). A carrier was defined as a patient without diarrhea whose rectal swab was positive for toxigenic *C. difficile*. CDI here was defined as diarrhea plus the presence of toxigenic *C. difficile* in stool, in the absence of other reasonable causes of diarrhea. Screening results were not shared with ICU clinicians and infection control nurses and no contact precautions were taken for carriers of toxigenic *C. difficile*.

### Screening, culture and PCR confirmation

Swabs and stool samples were processed immediately after arrival at the laboratory. Total DNA was prepared from swabs and stool using the Stool DNA Kit (OMEGA, Norcross, GA) and was screened for the species-specific gene *tpi* (encoding triose phosphate isomerase of *C. difficile*) and *tcdB* of *C. difficile* by PCR as described previously [7, 8].

Samples positive for both *tpi* and *tcdB* were cultured anaerobically. Briefly, samples were treated with an equal volume of 95 % ethanol before streaking onto cefoxitin cycloserine fructose agar (CCFA; OXOID, Basingstoke, UK) plates complemented with the CDMN supplement (OXOID) and 5 % sheep blood. Plates were incubated in anaerobic jars at 37 °C for 72 h. All isolates recovered were checked by morphological examination and aerotolerant experiments.

Genomic DNA was prepared from each isolate using the QIAamp DNA mini kit (Qiagen, Hilden, Germany) according to the manufacturer’s instructions for Gram-positive organisms. Isolates were confirmed as toxigenic *C. difficile* using PCR for *tpi* and *tcdB* [7, 8]. Toxigenic *C. difficile* isolates were also screened for the enterotoxin-encoding gene *tcdA* and binary toxin genes *cdtA* and *cdtB* using multiplex PCR [7, 8]. Isolates recovered were considered ICU-acquired if the host patient had no toxigenic *C. difficile* in the swab collected within 2 days of admission.

### Strain typing

Strain typing of toxigenic isolates was performed using multilocus sequence typing (MLST) as described previously [9]. Sequence types (STs) of *C. difficile* were also clustered into clades by phylogenetic analysis using the concatenated sequences of the seven loci of MLST [9]. For isolates belonging to the same ST, multiple-locus variable-number tandem repeat analysis (MLVA) was performed to further determine the relatedness of these isolates as described before [4]. The 6-loci MLVA scheme developed by Marsh J et al. [4, 10] was used and the exact size of amplicons was determined by capillary electrophoresis using an ABI 3730xl (Applied Biosystems, Carlsbad, CA).

### Statistical analysis

Statistical analysis was performed using SPSS version 18.0 (SPSS Inc., Chicago, IL). Proportions were compared using a Chi-Squared test and a *P* value <0.05 was considered statistically significant.

## Results

**Fig. 1:**
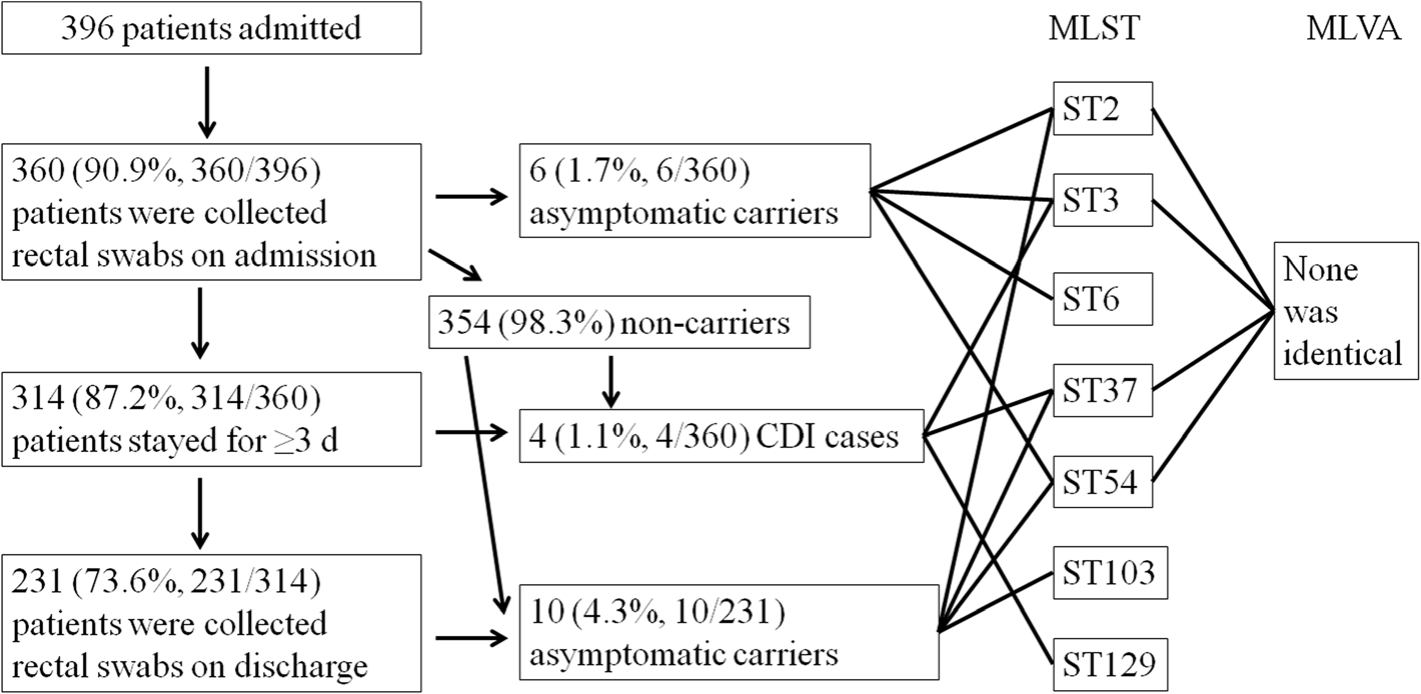
Summary of results in this study

During the 4-month period, a total of 396 adult patients were admitted to the ICU, among which rectal swabs were collected from 360 (90.9 %) patients within 2 days of admission to ICU (Fig. 1). Among the 360 patients, 314 had stayed in the ICU for at least 3 days, with rectal swabs collected from 231 (73.6 %, 231/314) patients within the 3 days prior to ICU discharge (Fig. 1). Only 6 (1.7 %, 6/360) patients had toxigenic *C. difficile* on admission, while 10 (4.3 %, 10/231) had it at discharge. As none developed diarrhea during their hospitalization in ICU, all 16 patients positive for toxigenic *C. difficile* (either on admission or at discharge) were classified as carriers. Of the 6 carriers of toxigenic *C. difficile* on admission, 4 had a rectal swab collected within 3 days of discharge but toxigenic *C. difficile* was not detected. For all 10 carriers of toxigenic *C. difficile* at discharge, swabs collected on admission were negative, suggesting that they acquired toxigenic *C. difficile* during their ICU stay. The prevalence of colonization with toxigenic *C. difficile* on admission appeared to be higher in patients who were 65 or older (3.3 %, 4/122) than that in those who were younger (0.8 %, 2/238) but this difference was not statistically significant (*P* = 0.2). Most (8/10) of patients acquired toxigenic *C. difficile* during their ICU stay were also 65 or older and had a higher rate of acquisition of toxigenic *C. difficile* (9.9 %, 8/81) than those who were younger (1.3 %, 2/150; *P* < 0.05).

Toxigenic *C. difficile* isolates were recovered from all of the 16 carriers detected by PCR. Among these 16 toxigenic isolates, 10 were positive for *tcdA* and *tcdB* (A + B+) and 6 were positive for *tcdB* only (A-B+). In addition, binary toxin genes were detected in one isolate, which was collected at discharge and was A + B+. The 6 isolates recovered from swabs collected on admission belonged to four STs, i.e. ST2 (*n* = 1), ST3 (*n* = 2), ST6 (*n* = 1) and ST54 (*n* = 2) (Fig. 1 and Table 1). In contrast, most (6 of 10) isolates recovered from swabs collected at discharge belonged to ST37, which was not detected on admission. The remaining four isolates recovered from swabs collected at discharge belonged to ST2 (*n* = 1), ST54 (*n* = 2) and ST103 (*n* = 1) (Fig. 1 and Table 1). The isolate carrying binary toxin genes belonged to ST103.

**Table 1:** Characteristics of patients with toxigenic *C. difficile*

Patient	ICU stay, d	Diseases	Collection date	ST	Clade^a^	MLVA profile^b^
Isolates detected on admission						
1	13	Cholangitis, bacteremia	November	2	1/HA1	316/171/264/280/280/371
2	19	Stroke	September	3	1/HA1	259/187/329/365/365/117
3	7	Systemic EB virus positive T lymphocyte proliferative disease, pneumonia	September	3	1/HA1	425/195/243/336/336/455
4	27	Pneumonia, Guillain-Barré syndrome	November	6	1/HA1	ND
5	6	Septic shock, colon cancer	August	54	1/HA1	322/171/286/365/365/117
6	4	Gastric cancer, pneumonia	September	54	1/HA1	322/171/286/344/344/223
Isolates detected on discharge						
7	12	Pneumonia, uremia	October	2	1/HA1	259/164/263/344/344/488
8	12	Embedded abdominal wall hernia	August	37	4/A-B+	129/336/209/314/314/420
9	6	Septic shock, ovian cancer	October	37	4/A-B+	129/344/209/294/293/438
10	10	Brain glioma	October	37	4/A-B+	129/336/209/307/307/421
11	17	Liver cirrhosis	November	37	4/A-B+	129/367/209/300/300/438
12	32	Pancreatitis	November	37	4/A-B+	129/336/209/300/300/487
13	5	Gangrenous appendicitis, peritonitis	November	37	4/A-B+	129/404/209/307/307/454
14	10	Pneumonia	August	54	1/HA1	276/171/294/365/365/117
15	29	Anterior pituitary hypofunction	October	54	1/HA1	218/187/294/414/414/117
16	8	Pneumonia	September	103	1/HA1	ND
Isolates causing CDI						
17	20	Pancreatitis	September	3	1/HA1	292/148/337/351/351/117
18	12	Colon cancer	September	37	4/A-B+	129/367/209/307/307/454
19	19	Liver cirrhosis	November	37	4/A-B+	129/367/209/300/300/420
20	29	Liver cirrhosis	September	129	1/HA1	ND

^a^Names of clades are from reference [9]. ^b^MLVA profile: amplicon size (bp) for loci CDA6, CDG8, CDR5, CDR48, CDR49 and CDR60

During their ICU stay, 54 (15.0 %) out of 360 patients developed diarrhea, of which four cases (1.1 %, 4/360; their ages ranged from 35 to 73) were CDI. The length of ICU stay was 3,744 ICU days for the 360 patients and CDI incidence was 10.7 cases per 10,000 ICU days. None of the four cases were positive for toxigenic *C. difficile* either on admission or at discharge. Toxigenic *C. difficile* was cultured from all four patients and belonged to ST3 (*n* = 1), ST37 (*n* = 2) or ST129 (*n* = 1) (Fig. 1 and Table 1).

In total, 20 toxigenic *C. difficile* isolates were recovered from either swabs or stool samples. The 20 isolates belonged to 7 STs (ST2, ST3, ST6, ST37, ST54, ST103 and ST129; Table 1). Toxigenic *C. difficile* could be clustered into four clades based on STs [9]. Among the 7 STs identified in this study, ST5 belongs to clade 3 and ST37 belongs to clade 4, while the remaining STs were of clade 1 (Table 1). As there were multiple isolates of ST2 (*n* = 2), ST3 (*n* = 2), ST37 (*n* = 8) and ST54 (*n* = 4), those belonging to the same ST were further typed using MLVA. However, none of these isolates had identical MLVA patterns (Fig. 1 and Table 1).

## Discussion

Previous studies revealed that the prevalence of colonization with toxigenic *C. difficile* in adult hospitalized patients varied from 4.4 % to 23.2 % [6] with the pooled rate being 8.1 % [5]. However, very few studies investigated the colonization of toxigenic *C. difficile* among ICU patients. In a study at Johns Hopkins Hospital, 3.1 % of patients were colonized with toxigenic *C. difficile* on admission to ICU. This suggests that the colonization rate of toxigenic *C. difficile* in ICU patients may be lower than those described for the general population due to as yet unknown reasons [11]. We cannot rule out that the low prevalence of colonization with toxigenic *C. difficile* on admission to ICU may be due to the use of a rectal swab rather than stool. However, prior studies have demonstrated that rectal swabs and stool cultures were equivalent for detection of *C. difficile* in stool of CDI patients [12, 13]. Nonetheless, the low prevalence (1.7 %) of colonization on admission to ICU in our study was even lower than the 3.1 % in study from Johns Hopkins Hospital, which also used rectal swabs for screening. The differences in colonization rate among studies remain unclear but may reflect the population risk of colonization with toxigenic *C. difficile* as previous studies have demonstrated that the prevalence of colonization with toxigenic *C. difficile* varies significantly by geographic location [6]. Although the prevalence of toxigenic *C. difficile* in China remains still largely unknown, it is possible that the prevalence of colonization with toxigenic *C. difficile* in China is truly lower than that in western countries. This may not be a surprise as there are significant differences in food and drinking habits between China and western countries. However, large-scale multi-center studies are required to fully characterize the prevalence of toxigenic *C. difficile* in China.

A systematic review and meta analysis found that patients colonized with toxigenic *C. difficile* on hospital admission were 6 times more likely to develop CDIs compared with noncolonized patients [5]. In the previous study on ICU patients, colonization with toxigenic *C. difficile* on admission and colonization during ICU stay have also been identified as an independent risk factors for subsequent CDI with relative risks being 8.62 and 10.93, respectively [11]. However, it has also been thought that colonization with *C. difficile* could be protected from subsequent CDI [14]. In this study, none of the six patients colonized with toxigenic *C. difficile* on admission and none of the ten patients colonized at discharge developed diarrhea during their ICU stay. Patients who were 65 years of age or older had a much higher risk of developing CDI than younger patients [15]. Our study found that patients who were 65 or older were also more likely to acquire toxigenic *C. difficile* and become asymptomatic carriers during their ICU stay. Active screening of elderly patients was therefore able to identify more carriers of toxigenic *C. difficile* and was more resource-efficient than screening all patients. However, the significance of active screening in clinical management and infection control needs to be justified as none of these elderly carriers developed CDI in our setting. Our findings do not support the active screening of all patients for toxigenic *C. difficile* on admission to ICU, in the setting of a low prevalence of colonization, to identify patients in high risks of subsequent CDI. More studies are warranted to confirm whether colonization with toxigenic *C. difficile* is truly a high-risk factor for subsequent CDI in ICU patients.

None of the four patients colonized with a toxigenic *C. difficile* had CDI at discharge. A previous study reported that β-lactam-β-lactamase inhibitor combinations and metronidazole were associated with a loss of *C. difficile* colonization [16]. During their ICU stay, three of the four patients received either piperacillin-tazobactam (*n* = 2) or cefoperazone-sulbactam (*n* = 1) and the remaining patient received metronidazole.

Ten patients had toxigenic *C. difficile* at discharge and four patients developed CDI during their ICU stay. The fourteen patients did not have toxigenic *C. difficile* on admission, suggesting that their toxigenic *C. difficile* were acquired during their ICU stay. An obvious concern is whether these ICU-acquired isolates were from those introduced to ICU on admission. However, the majority (10/14) of ICU-acquired toxigenic *C. difficile* belonged to STs, which were not detected on admission. The remaining four ICU-acquired isolates (2 of ST54, 1 of ST2 and 1 of ST3) matched those detected on admission with the same ST. However, MLVA analysis revealed that none of these paired ICU-acquired and introduced isolates were identical. Therefore, there was no evidence that the ICU-acquisitions were from those introduced isolates. This unexpected finding suggests that there were additional sources (e.g. healthcare workers, visiting family members or the environment) of toxigenic *C. difficile* in ICU, which is yet to be determined. Our findings do not support the active screening of all patients for toxigenic *C. difficile* on admission to ICU in our setting to identify the potential source of subsequent CDI. In a setting with low prevalence of colonization, active screening for toxigenic *C. difficile* may not be justified as a resource-efficient measure for the prevention of CDI.

Most of the ICU-acquired toxigenic *C. difficile* isolates (8/14) belonged to a single ST, ST37, suggesting possible transmission of a certain clone in ICU. However, none of the 8 ST37 isolates were identical by MLVA, which does not support the hypothesis of the transmission of a certain clone. Alternatively, although MLVA has been widely adopted for strain typing and has been successfully used for *C. difficile* [4], it may be too discriminatory to define clones, which has been observed by other investigators [17–19]. It is therefore possible that MLVA may not be an ideal method for investigating the transmission of toxigenic *C. difficile*. Molecular typing is critical to track the transmission of toxigenic *C. difficile* within hospitals but none of the currently-used typing methods, including pulsed field gel electrophoresis (PFGE), restriction enzyme analysis (REA), MLST and PCR ribotyping, is ideal for *C. difficile* [20]. Although a previous study found that MLVA and whole genome sequencing had similar discriminatory ability [21], whole genome sequencing may still be required to untangle whether there was clonal transmission of toxigenic *C. difficile* in ICU.

We found a binary toxin-producing *C. difficile* isolate, which colonized (rather than caused CDI) a patient on admission to ICU. Recent publications showed that binary toxin is a marker for highly virulent *C. difficile* or contributes directly to the virulence [22]. The identification of a colonizing binary toxin-positive isolate in this study highlights that the presence of binary toxin genes does not necessarily indicate diarrhea, which also depends on host factors.

CDI incidence (10.7 cases per 10,000 ICU days) in this study was lower than that (25.2 cases per 10,000 ICU days) of our previous study, which was performed between May 2012 and January 2013 in the same unit [23]. This could be due to improved infection control practices in the unit. For instance, hand hygiene compliance of healthcare workers in the unit had increased from 49.5 % in 2012 to 79.7 % in 2014. Ongoing monitoring of CDI incidence is required to examine whether the reduction of CDI continues.

Our study has several limitations. First, this was a single center study and we were not able to collect swabs from all eligible patients and some additional carriers might have been missed. Second, this study was carried out to explore the prevalence of colonization with and the transmission of toxigenic *C. difficile* in ICU, we did not specifically investigate the risk factors for the acquisition of toxigenic *C. difficile* in ICU. In addition, the small number of patients colonized with toxigenic *C. difficile* may result in our study being underpowered for the detection of risk factors for colonization. Third, we did not follow up the carriers who were positive at discharge from ICU and therefore we were unable to find out whether these colonizers developed diarrhea later on. Without follow up we were also unable to determine whether the colonization of toxigenic *C. difficile* was transient or could persist for a period. Fourth, as previous studies have demonstrated that PCR assays may be less sensitive than toxigenic culture for detection of asymptomatic carriage of toxigenic C. difficile [12, 13], some carriers might have been missed in this study.

## Conclusions

The prevalence of colonization with toxigenic *C. difficile* and the incidence of CDI among patients on admission to ICU were low in our settings. Although some patients (4.3 %) acquired toxigenic *C. difficile* and became asymptomatic carriers during their ICU stay, these ICU-acquired isolates were not genetically linked with those carried by patients at admission. Active screening for toxigenic *C. difficile* may not be a resource-efficient measure for the prevention of CDI in such a setting with a low prevalence of colonization. Importantly, these conclusions may be not generalizable due to the low prevalence of *C. difficile* colonization and CDI in our patient population.

## Abbreviations

CDI, *C. difficile* infection; MLST, multi-locus sequence typing; MLVA, multiple-locus variable number tandem repeat analysis; ST, sequence type
